# Network meta-analysis of different acupuncture methods for post-stroke upper-limb spasticity

**DOI:** 10.3389/fneur.2026.1725618

**Published:** 2026-02-10

**Authors:** Fanyu Xing, Rongwei Zhang, Yang Guo, Yanfei Wang, Yuhong Guo, Linxuan Yang, Zhaoqing Wang, Zhitong Liu, Ruixing Jiang, Jingyi Wang, Yiting Zhao, Jiangwei Shi, Lili Yin

**Affiliations:** 1 Tianjin University of Traditional Chinese Medicine, Tianjin, China; 2 First Teaching Hospital of Tianjin University of Traditional Chinese Medicine,National Clinical Research Center for Chinese Medicine, Tianjin, China; 3 The Second Affiliated Hospital of Tianjin University of Traditional Chinese Medicine, Tianjin, China; 4 Baoshan Hospital of Traditional Chinese Medicine, Baoshan, China

**Keywords:** acupuncture, network meta-analysis, spasticity, stroke, upper limb

## Abstract

**Introduction:**

Currently, acupuncture therapy is widely used for post-stroke upper-limb spasticity. However, the available evidence remains insufficient to determine the relative effectiveness of different acupuncture protocols.

**Methods:**

This study retrieved relevant databases and systematically reviewed randomized controlled trials (RCTs) on acupuncture treatment for post-stroke upper-limb spasticity. A total of 28 trials involving 14 acupuncture treatment protocols were included. A network meta-analysis was performed using Stata 18 software.

**Results:**

The results indicated that the Balanced Yin-Yang Acupuncture + Rehabilitation yielded the best outcomes in improving Fugl–Meyer Assessment scores, while Luan’s Three-Needle Technique combined with Meridian Sinew Cluster Needling + Rehabilitation was most effective in reducing modified Ashworth scale scores.

**Discussion:**

A comparison of efficacy indicators across 14 different acupuncture methods combined with rehabilitation showed that Balanced Yin-Yang Acupuncture + Rehabilitation and Luan’s Three-Needle Technique combined with Meridian Sinew Cluster Needling + Rehabilitation were more effective in treating post-stroke upper-limb spasticity. Owing to limitations in the current body of research, these conclusions need to be further verified by more high-quality randomized controlled trials.

**Systematic review registration:**

https://www.crd.york.ac.uk/PROSPERO, identifier CRD420251110982.

## 1 Introduction

Post-stroke spastic paralysis is one of the most common disabling complications in stroke patients. Approximately 43% of stroke survivors experience spasticity within 12 months of the acute episode, and the prevalence increases to as high as 97% in the chronic phase (1). With the annual increase in the global number of stroke cases, post-stroke spastic paralysis has become a core issue leading to motor dysfunction and reduced quality of life in patients (2). Spasticity is a motor disorder characterized by a velocity-dependent increase in tonic stretch reflexes, elevated muscle tone, and hyperactive tendon reflexes (3). Upper-limb spasticity often manifests itself as shoulder adduction and internal rotation, accompanied by elbow flexion, wrist flexion, and finger flexion (4). Modern medical interventions primarily include oral antispastic medications, botulinum toxin injections, and rehabilitation therapy. However, these approaches often involve strong drug dependency and are prone to adverse reactions such as fatigue and drowsiness (5). Acupuncture, as a traditional Chinese medicine therapy, has shown considerable efficacy in treating post-stroke spasticity (6). Among various acupuncture techniques, filiform needle acupuncture is a fundamental clinical intervention due to its simplicity, high safety, and low cost. Nevertheless, the wide diversity of acupuncture protocols precludes a definitive conclusion regarding which approach is most effective for post-stroke upper-limb spasticity. Network meta-analysis (NMA) can simultaneously compare the effects of multiple interventions and rank the efficacy of each (7). This study aims to use NMA to evaluate the therapeutic effects of different acupuncture methods to identify the optimal treatment, thereby providing evidence-based support for clinical decision-making.

## 2 Methods

This study was conducted following the Preferred Reporting Items for Systematic Reviews and Meta-Analyses (PRISMA) reporting guidelines and has been registered on the PROSPERO platform under registration number CRD420251110982. Clinical trial number: not applicable.

### 2.1 Inclusion criteria

The inclusion criteria were as follows: ① Study type: Randomized controlled trials (RCTs) on filiform needle acupuncture for post-stroke spasticity.

② Participants: Patients meeting the diagnostic criteria for stroke and presenting clinical features such as increased muscle tone, abnormal tendon reflexes, and clonus.

③ Interventions: The control group received rehabilitation alone. The treatment group received filiform needle acupuncture in addition to rehabilitation. In both groups, conventional internal medicine treatments for underlying diseases were permitted.

④ Primary outcome measures: (a) Fugl–Meyer Assessment (FMA) scores (b) Modified Ashworth Scale (MAS). The included trials were required to report at least one outcome measure related to upper-limb spasticity.

### 2.2 Exclusion criteria

The exclusion criteria were as follows: ① Duplicate publications, for which only the most recent study was included. ② Trials with incomplete data and or unavailable full texts. ③ Reviews, animal experiments, conference proceedings, dissertations, and experience summaries. ④ Trials involving the use of muscle relaxants. ⑤ Trials in which outcome measures did not distinguish between upper and lower limbs.

### 2.3 Search strategy

A comprehensive computerized search was conducted across multiple electronic databases, including China National Knowledge Infrastructure (CNKI), Wanfang Database, VIP Database (VIP), China Biology Medicine disc (CBM), PubMed, Embase, Cochrane Library, and Web of Science. The search strategy used a combination of subject headings and free-text terms. The search terms included stroke, cerebral infarction, cerebral hemorrhage, spasm, spasticity, spastic paralysis, muscle tonus, randomized controlled trials, RCT, and acupuncture. The search encompassed all records from inception to October 2025. Detailed information on the literature search terms can be found in Supplementary file 1.

### 2.4 Literature retrieval and data extraction

Literature screening and review were conducted independently by two researchers. Discrepancies were resolved through discussion or, when necessary, consultation with a third researcher. After a consensus was reached, data were extracted, including the following primary information: first author, publication date, sample size, mean age, treatment measures and course, outcome indicators, and elements for risk bias assessment.

### 2.5 Literature quality assessment

The Cochrane Risk of Bias tool was used to assess the included trials (8). The assessment covered the following aspects: random sequence generation, allocation concealment, blinding of participants and personnel, blinding of outcome assessment, completeness of outcome data, selective reporting, and other potential biases.

### 2.6 Statistical analysis

RevMan 5.4 software was used to generate the literature quality assessment graph. Stata 18 software was used for data analysis. For continuous variables, the mean difference (MD) was selected as the effect size. A *p*-value of < 0.05 was considered statistically significant. Stata 18 software was used to construct the network evidence graph, in which each node represents an intervention, the size of the node corresponds to the number of cases for that intervention, and the solid lines between nodes indicate direct comparison between two interventions, with the thickness of the line reflecting the amount of direct comparison evidence. If the network evidence graph did not form closed loops, the consistency model was used for the NMA. If closed loops were present, the node-splitting method was applied for inconsistency testing. The surface under the cumulative ranking curve (SUCRA) was calculated to rank the efficacy of various interventions. A comparison-adjusted funnel plot was generated using Stata 18 software to assess publication bias and small-study effects within the intervention network.

## 3 Results

### 3.1 Literature search results

**Figure 1:**
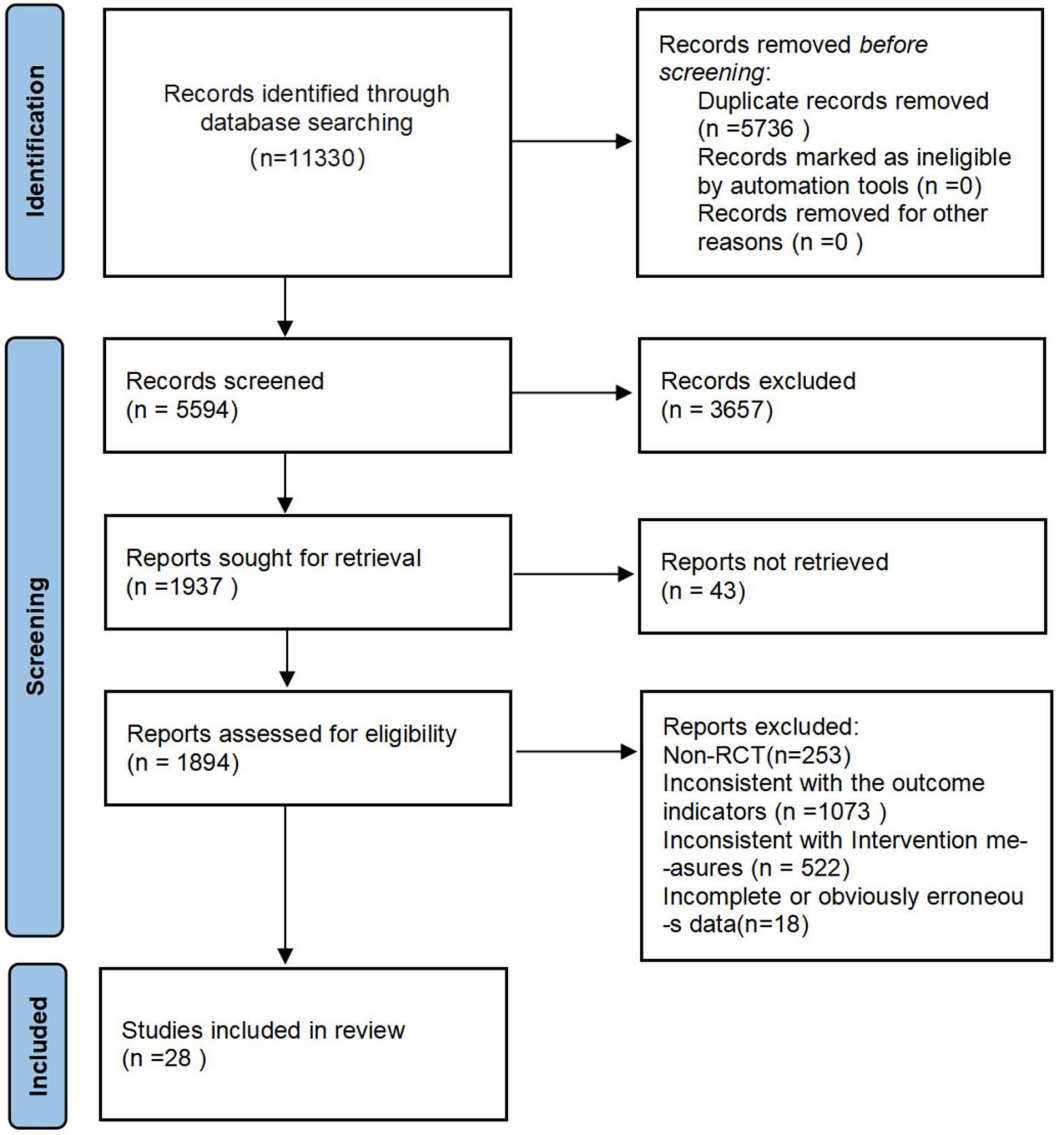
Screening flow diagram.

The initial search retrieved 11,330 articles (10,634 in Chinese and 696 in English). After removing 5,736 duplicates, 5,594 articles remained for initial screening. Following a detailed review of abstracts and full texts, 28 articles were ultimately included. The detailed screening process is shown in Figure 1.

### 3.2 Basic characteristics of included trials

The 28 included trials involved a total of 2,626 patients, of which 1,317 were in treatment groups and 1,309 in control groups. All included trials were two-arm trials. They involved 14 acupuncture methods, including: Governor Vessel Acupuncture + Rehabilitation, Xingnao Kaiqiao Acupuncture + Rehabilitation, Xingnao Kaiqiao Acupuncture combined with Scalp Acupuncture + Rehabilitation, Scalp Acupuncture + Rehabilitation, Jin’s Three-Needle Technique + Rehabilitation, Luan’s Three-Needle Technique combined with Meridian Sinew Cluster Needling + Rehabilitation, Penetrating Needling + Rehabilitation, Meridian Sinew Acupuncture + Rehabilitation, Acupuncture at Shangjiejing Point Plus Jing-Well Points + Rehabilitation, Balanced Yin-Yang Acupuncture + Rehabilitation, Acupuncture at Jiaji Points + Rehabilitation, Acupuncture at Antagonist Muscles + Rehabilitation, Scalp Acupuncture combined with Yangming Meridian Acupuncture + Rehabilitation, and Yangming Meridian Acupuncture + Rehabilitation. The baseline characteristics of the included trials are presented in Table 1.

**Table 1:** Characteristics of included trials.

Author	Year	Treatment group			Control group			Treatment duration (days)	Outcome measures
		*n*	Intervention	Mean age	*n*	Intervention	Mean age		
Zhang Junyu	2023	32	B	68 ± 8	31	A	66 ± 9	28	①②
Zhang Zhixin	2024	40	B	63.92 ± 4.14	40	A	63.86 ± 4.17	42	①
Li Guanglin	2025	30	B	58.49 ± 5.8	30	A	58.96 ± 5.9	28	②
Yang Man	2018	42	C	65.53 ± 4.19	42	A	65.59 ± 4.24	28	①
Liu Hongjie	2023	30	C	55.23 ± 7.86	29	A	54.83 ± 13.92	30	①②
Lou Anhua	2023	65	C	54.76 ± 9.98	60	A	53.68 ± 9.25	14	①
Ma Xiaoli	2023	53	C	61.83 ± 8.18	53	A	62.76 ± 7.36	28	②
Du Liangbin	2023	31	D	63.12 ± 8.45	34	A	62.26 ± 8.26	90	①
Wang Zhihong	2023	90	D	35 ~ 85	90	A	35 ~ 85	56	①②
Jin Lihui	2023	46	E	59.48 ± 9.16	46	A	58.72 ± 8.93	28	①
Qi Lili	2018	30	E	64 ± 10	30	A	65 ± 9	30	②
Lv Lili	2022	51	F	63.15 ± 4.19	51	A	62.08 + 4.32	28	①
Lang Jianying	2013	47	F	65 ± 9	47	A	64 ± 9	28	①
Xu Shifen	2016	36	F	60 ± 10	35	A	65 ± 6	28	①
Ye Weibin	2019	60	G	67.3 ± 3.4	60	C	62.3 ± 2.1	20	②
Zhu Jinmei	2020	30	H	63 ± 10	30	A	64 ± 13	28	①②
Tan Shihong	2018	44	H	54.93 ± 7.82	44	A	54.78 ± 7.69	28	①②
Wen Hongyuan	2022	41	I	53.38 ± 4.69	40	A	53.25 ± 4.70	28	①②
Ni Huanhuan	2012	50	J	40–79	50	A	40–79	28	①
Chen Hailing	2020	60	J	62.53 ± 2.14	60	A	62.45 ± 2.18	28	①
Hu Yinghua	2017	45	K	55.30 ± 5.20	45	A	54.80 ± 4.90	14	①②
Chen Lijun	2020	39	K	53.7 ± 4.4	39	A	53.5 ± 4.1	14	①②
Wang Ya	2021	58	K	58.46 ± 6.71	58	A	58.32 ± 5.24	30	①②
Liao Mingxuan	2018	37	L	51.5 ± 4.65	35	A	50.9 ± 5.65	28	①
Zhao Juanjuan	2021	71	L	65.65 ± 8.15	71	A	65.62 ± 8.11	56	①
Cao Qinning	2012	30	M	62.51 ± 7.18	30	A	61.74 ± 7.68	75	①②
Qiu Lin	2014	45	N	60.2 ± 10.4	45	A	59.6 ± 9.7	56	①
Xia Zhaoxin	2018	84	P	67 ± 5.99	84	A	68 ± 5.43	21	

A: Rehabilitation only, B: Governor Vessel Acupuncture + Rehabilitation, C: Xingnao Kaiqiao Acupuncture + Rehabilitation, D: Xingnao Kaiqiao Acupuncture combined with Scalp Acupuncture + Rehabilitation, E: Scalp Acupuncture + Rehabilitation, F: Jin’s Three-Needle Technique + Rehabilitation, G: Luan’s Three-Needle Technique combined with Meridian Sinew Cluster Needling + Rehabilitation, H: Penetrating Needling + Rehabilitation, I: Meridian Sinew Acupuncture + Rehabilitation, J: Acupuncture at Shangjiejing Point Plus Jing-Well Points + Rehabilitation, K: Balanced Yin-Yang Acupuncture + Rehabilitation, L: Acupuncture at Jiaji (EX-B2) Points + Rehabilitation, M: Acupuncture at Antagonist Muscles + Rehabilitation, N: Scalp Acupuncture combined with Yangming Meridian Acupuncture + Rehabilitation, and O: Yangming Meridian Acupuncture + Rehabilitation. Outcome measures: ① FMA and ② MAS. Detailed information for each individual study can be found in Supplementary file 2, which includes specific scoring of the outcome measures.

### 3.3 Literature quality assessment

**Figure 2:**
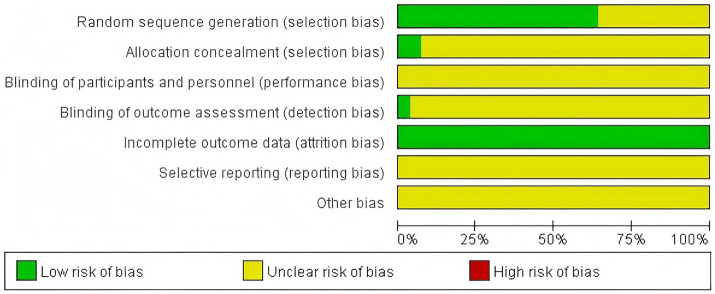
Risk of bias.

- Randomization method: Among the 28 included trials, 17 trials (9–25) used a random number table and 1 trial (26) used random drawing, which were rated as low risk. Nine trials (27–35) only mentioned “randomization” without any specification, and one trial (36) only referred to computer grouping without a detailed description; these were rated as unclear risk.
- Allocation concealment: Two trials (10, 18) adopted sealed-envelope methods and were therefore rated as low risk. The remaining trials did not mention whether allocation concealment was implemented and were rated as unclear risk.
- Blinding: None of the trials described blinding procedures or implementation details. One trial (10) reported blinding of outcome assessors and was rated as low risk, whereas the remaining trials did not specify the method of outcome assessment and were rated as unclear risk.
- Incomplete outcome data: Four trials (10, 12, 24, 29) reported data attrition, but dropout rates were low and unlikely to affect the intervention effect estimate; thus, they were rated as low risk.
- Selective reporting: None of the included trials provided protocols or pre-registered plans, making it impossible to assess selective reporting risk; all were rated as unclear risk.
- Other biases: Sources of other potential biases were unclear across all trials, resulting in an unclear risk rating. The risk of bias assessment results for the included trials are presented in Figure 2.

### 3.4 Network geometry

**Figure 3:**
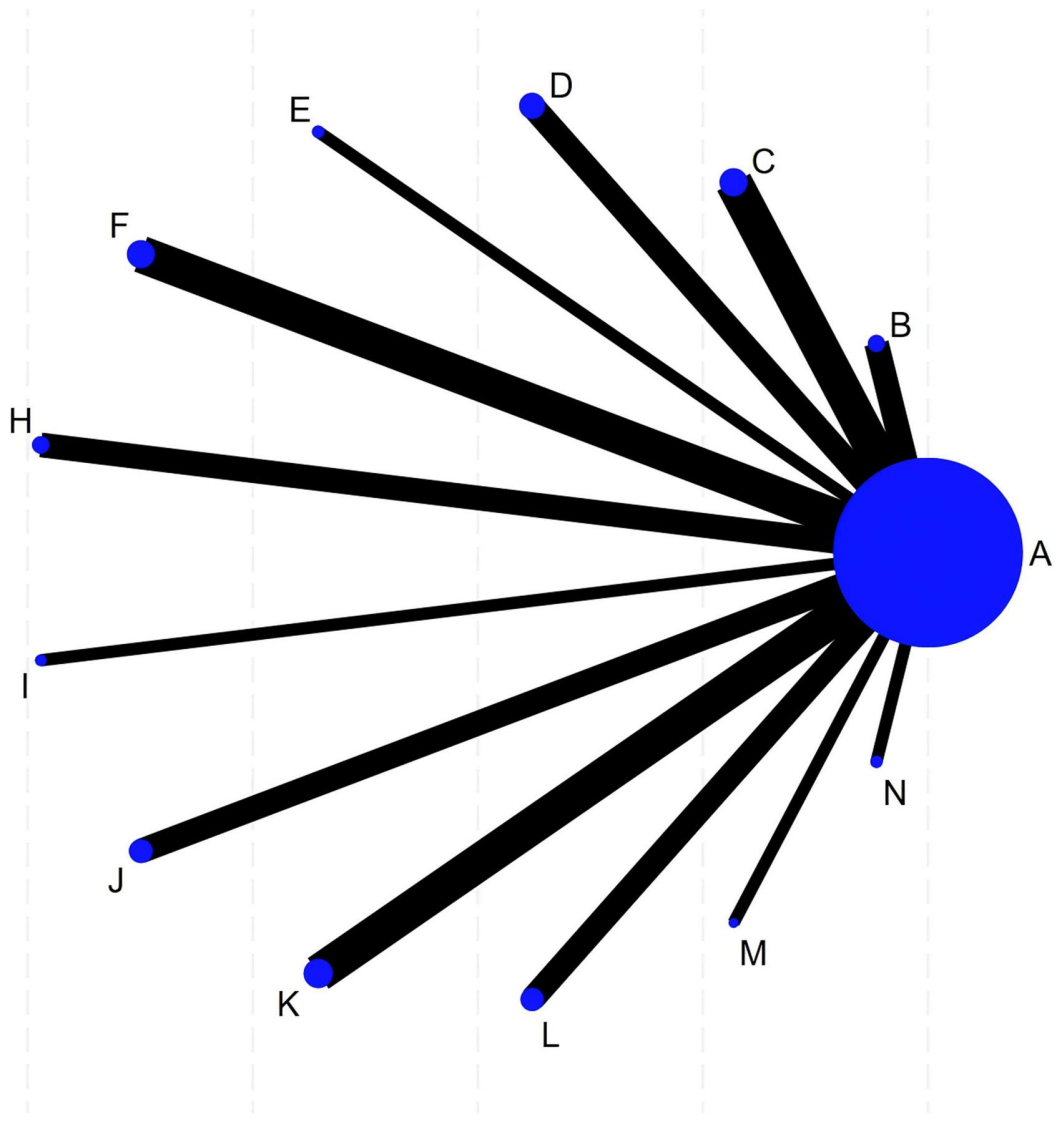
Network evidence graph of FMA.

**Figure 4:**
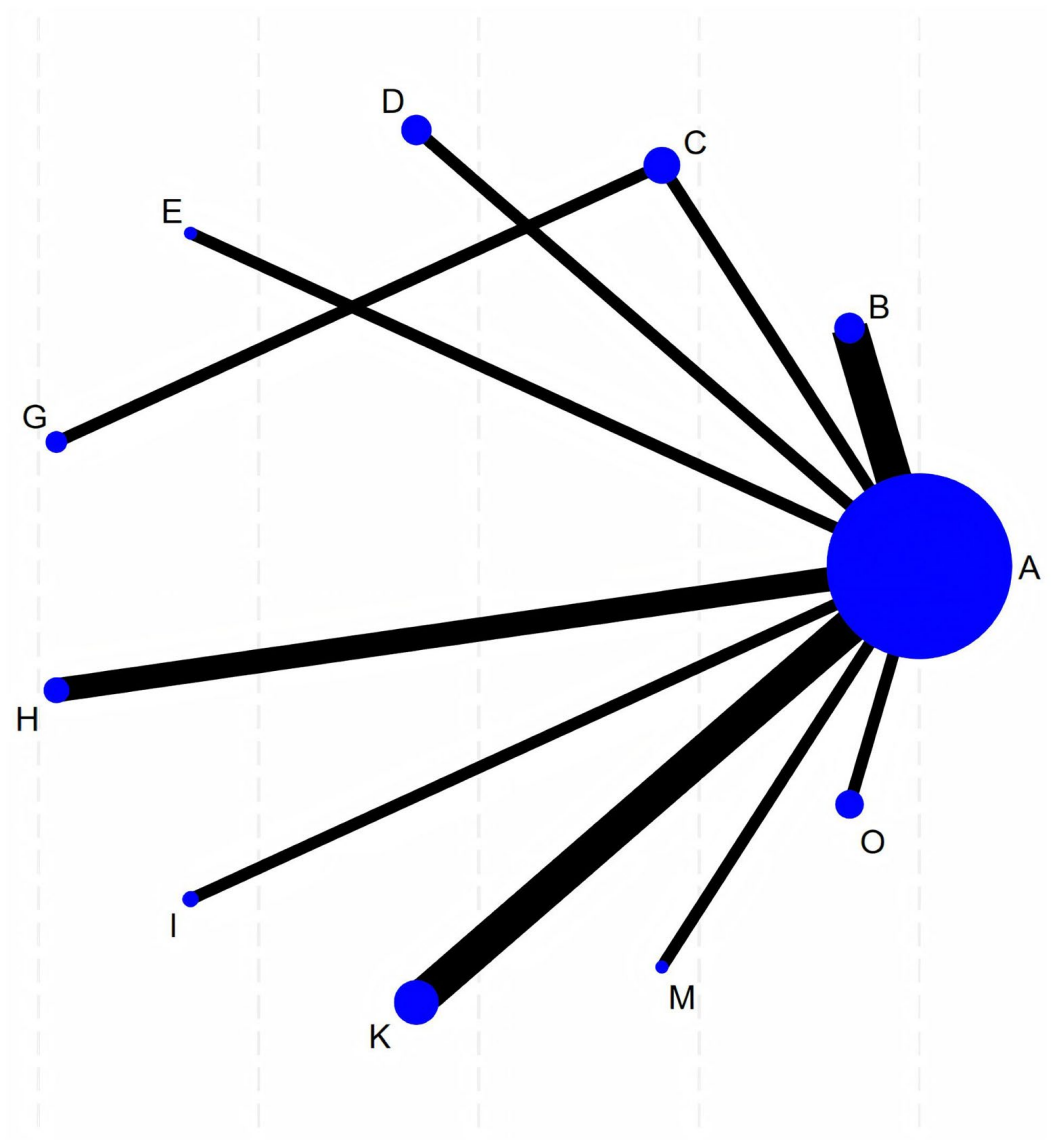
Network evidence graph of MAS. In the figures above, each lettered node represents an intervention (the corresponding acupuncture method for each letter is shown in Table 1). Both network evidence graphs feature A (rehabilitation) as the central node. The size of a node is proportional to the total sample size of that intervention. Lines connecting nodes indicate the existence of RCTs with direct comparisons between the interventions, and the thickness of a line is proportional to the number of studies providing direct comparative evidence. The network geometry indicates that most interventions lack direct comparative evidence, and the efficacy evaluation relies heavily on indirect comparisons using rehabilitation training as a common comparator.

Twenty-three trials reported the FMA involving 12 different acupuncture methods. The network graph for FMA is shown in Figure 3. Fifteen trials reported MAS, involving 10 different acupuncture methods. The network graph for MAS is shown in Figure 4. No closed loops were formed between interventions, making inconsistency testing unnecessary.

### 3.5 Network meta-analysis results

For FMA, the following interventions combined with rehabilitation were superior to rehabilitation alone (*p* < 0.05): Xingnao Kaiqiao Acupuncture [MD = 6.74, 95%CI (2.13, 11.35)], Xingnao Kaiqiao Acupuncture combined with Scalp Acupuncture [MD = 6.33, 95%CI (0.38, 12.28)], Jin’s Three-Needle Technique [MD = 8.47, 95%CI (3.33, 13.61)], Acupuncture at Shangjiejing Point Plus Jing-Well Points [MD = 6.17, 95%CI (0.83, 11.50)], Balanced Yin-Yang Acupuncture [MD = 10.63, 95%CI (6.06, 15.21)], and Acupuncture at Jiaji Points [MD = 7.60, 95%CI (2.07, 13.12)]. No statistically significant differences were observed in pairwise comparisons between the other interventions.

**Figure 5:**
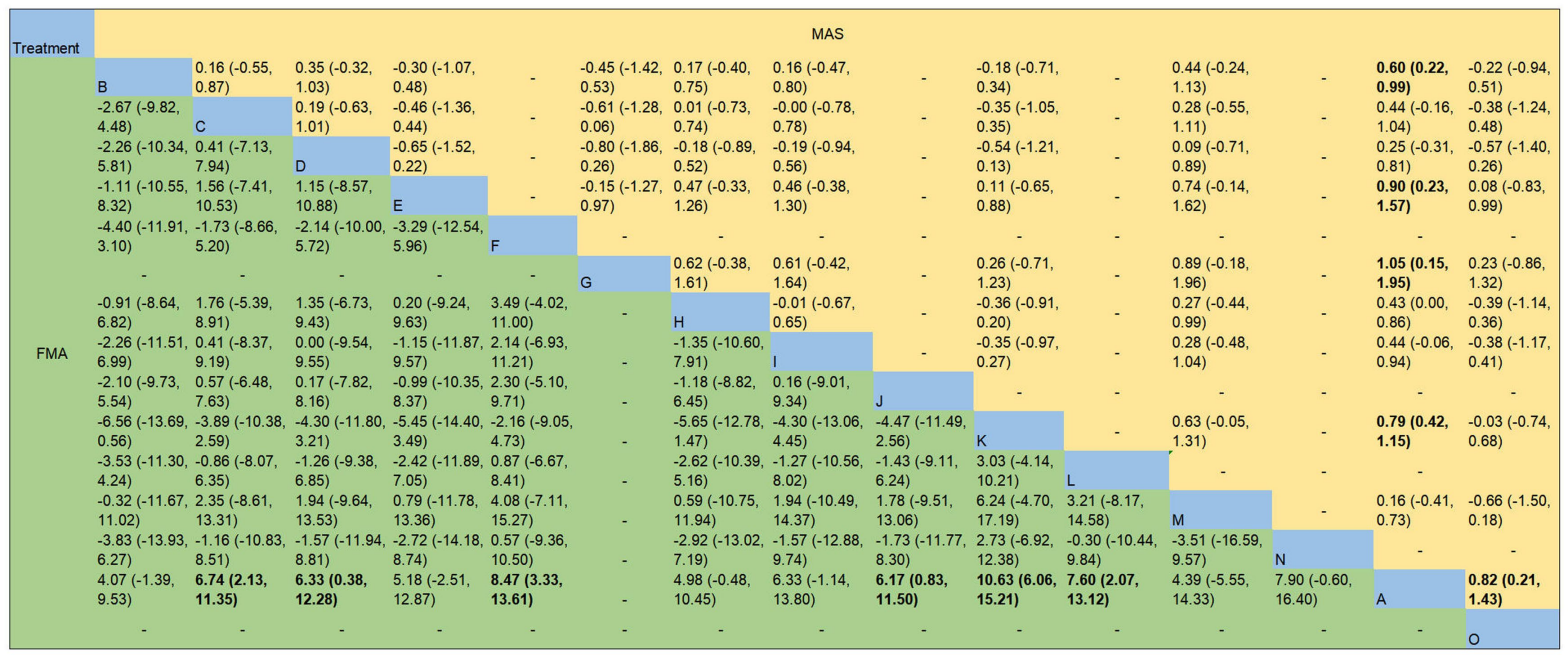
Network meta-analysis results for FMA and MAS. The figures display the mean differences and 95% confidence intervals for pairwise comparisons between interventions. When the confidence interval includes 0, it indicates that the difference is not statistically significant. Bold type denotes statistical significance. The corresponding acupuncture treatment methods for the English letters are as described in the notes of Table 1.

For MAS, the following interventions combined with rehabilitation were superior to rehabilitation alone (*p* < 0.05): Governor Vessel Acupuncture [MD = -0.60, 95%CI (−0.99, −0.22)], Scalp Acupuncture [MD = -0.90, 95%CI (−1.57, −0.23)], Luan’s Three-Needle Technique combined with Meridian Sinew Cluster Needling [MD = -1.05, 95%CI (−1.95, −0.15)], Balanced Yin-Yang Acupuncture [MD = -0.79, 95%CI (−1.15, −0.42)], and Yangming Meridian Acupuncture [MD = -0.82, 95%CI (−1.43, −0.21)]. No statistically significant differences were observed in pairwise comparisons between the other interventions. Specific results are shown in Figure 5.

### 3.6 SUCRA ranking

Interventions were ranked from best to worst based on SUCRA values for FMA: Balanced Yin-Yang Acupuncture + Rehabilitation (86.3%) > Jin’s Three-Needle Technique + Rehabilitation (70.0%) > Scalp Acupuncture combined with Yangming Meridian Acupuncture + Rehabilitation (63.1%) > Acupuncture at Jiaji Points + Rehabilitation (62.5%) > Xingnao Kaiqiao Acupuncture + Rehabilitation (55.5%) > Xingnao Kaiqiao Acupuncture combined with Scalp Acupuncture + Rehabilitation (51.8%) > Meridian Sinew Acupuncture + Rehabilitation (51.7%) > Acupuncture at Shangjiejing Point Plus Jing-Well Points + Rehabilitation (50.3%) > Scalp Acupuncture + Rehabilitation (43.2%) > Penetrating Needling + Rehabilitation (39.8%) > Acupuncture at Antagonist Muscles + Rehabilitation (38.4%) > Governor Vessel Acupuncture + Rehabilitation (32.9%) > Rehabilitation alone (4.4%).

Interventions were ranked from best to worst based on SUCRA values for modified Ashworth scores: Luan’s Three-Needle Technique combined with Meridian Sinew Cluster Needling + Rehabilitation (83.7%) > Scalp Acupuncture + Rehabilitation (78.0%) > Balanced Yin-Yang Acupuncture + Rehabilitation (73.9%) > Yangming Meridian Acupuncture + Rehabilitation (73.7%) > Governor Vessel Acupuncture + Rehabilitation (57.6%) > Meridian Sinew Acupuncture + Rehabilitation (43.4%) > Penetrating Needling + Rehabilitation (42.2%) > Xingnao Kaiqiao Acupuncture + Rehabilitation (41.8%) > Xingnao Kaiqiao Acupuncture combined with Scalp Acupuncture + Rehabilitation (27.9%) > Acupuncture At Antagonist Muscles + Rehabilitation (21.3%) > Rehabilitation alone (6.5%).

### 3.7 Publication Bias analysis

**Figure 6:**
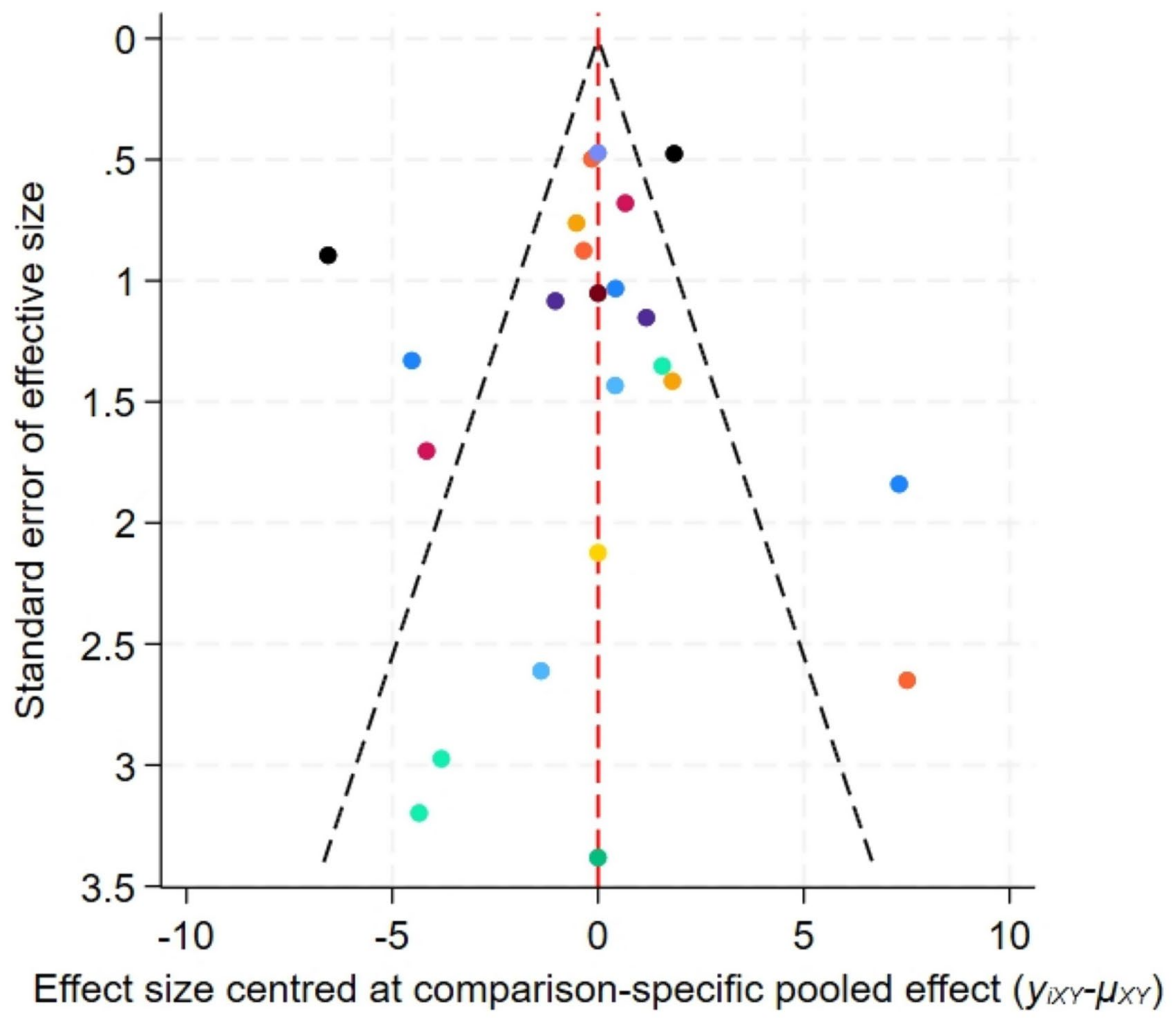
Funnel plot for the network meta-analysis of FMA.

**Figure 7:**
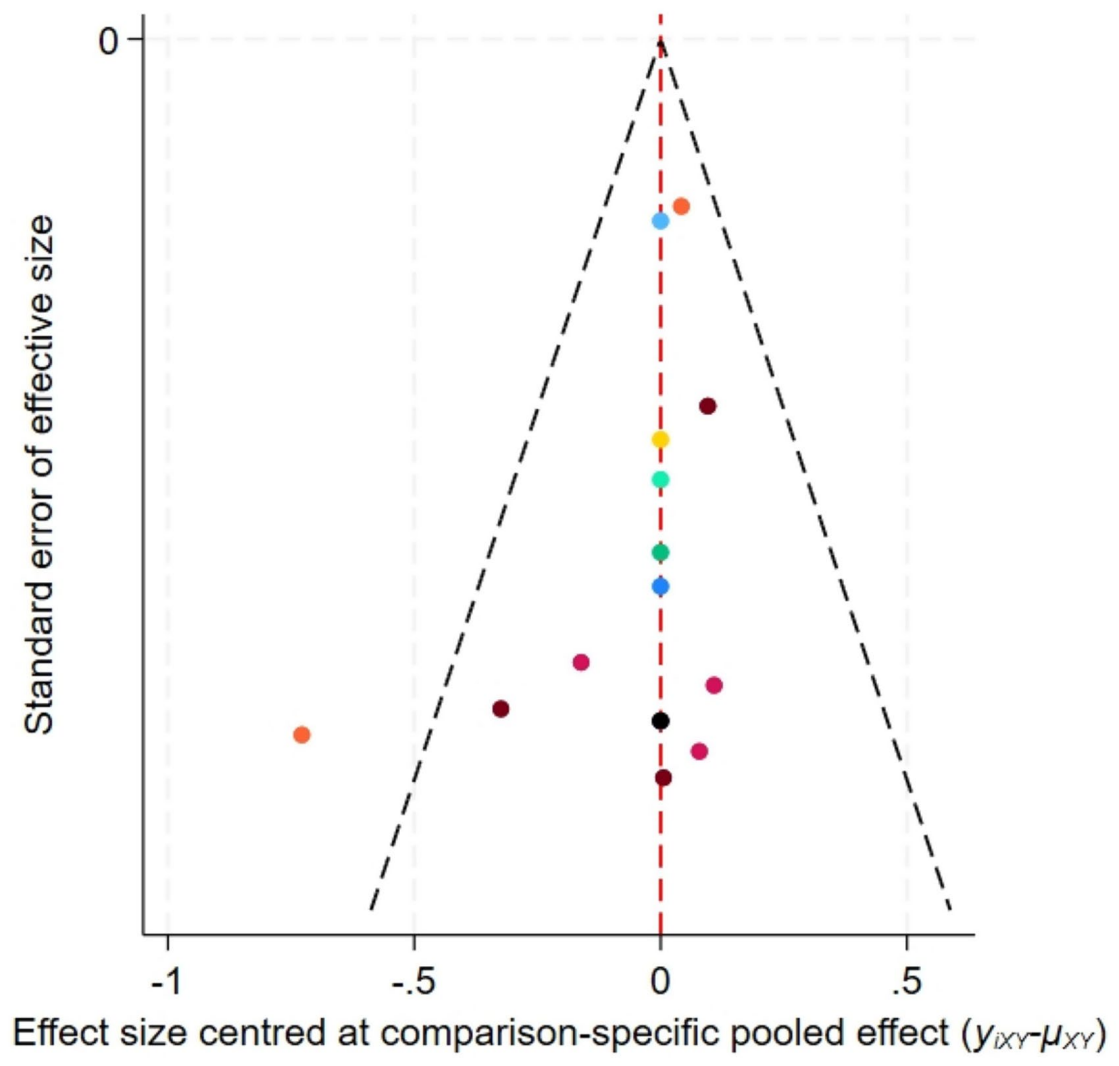
Funnel plot for the network meta-analysis of MAS.

Figure 6 displays the funnel plot for FMA, while Figure 7 presents the corresponding funnel plot for MAS. The distribution pattern demonstrates moderate symmetry, with several studies located beyond the funnel plot’s confidence limits, indicating potential publication bias or influences from small-sample effects.

## 4 Discussion

This study systematically evaluated the interventional effects of 14 acupuncture methods on post-stroke upper-limb spasticity using NMA. The NMA results indicated that Balanced Yin-Yang Acupuncture + Rehabilitation was the most effective in improving FMA scores. Balanced Yin-Yang Acupuncture uses point selection from both the medial and lateral aspects of the upper limb. For spastic muscle groups, shallow needling is applied, followed by rapid, small-amplitude lifting and thrusting with a reducing technique after obtaining qi. For antagonist muscle groups, appropriately deep needling is applied, followed by uniform, slow twisting with a reinforcing technique after obtaining qi. Modern studies suggest that Balanced Yin-Yang acupuncture can improve serum levels of transforming growth factor-β1 (TGF-β1) and neuron-specific enolase (NSE) in patients, which may help improve the prognosis of post-stroke spasticity (37). Furthermore, evidence from (24) indicates that acupuncture can effectively activate stretch receptors such as Golgi tendon organs, inhibit *α* motor neurons innervating spastic muscles, and simultaneously excite motor neurons of antagonist muscles. Through these mechanisms, acupuncture achieves the goal of inhibiting spastic muscles and activating antagonist muscles, thereby harmonizing limb muscle tone and restoring normal movement patterns.

Regarding the reduction of MAS scores, Luan’s Three-Needle Technique combined with Meridian Sinew Cluster Needling + Rehabilitation ranked first. The acupoints selected in Luan’s Three-Needle Technique are often adjacent to the main nerve trunks of the body, focusing on local points around the spastic joints. The acupoint selection for Luan’s Three-Needle Technique in the upper limb includes Neiguan (PC 6), Jiquan (HT 1), and Chize (LU 5). Modern research has found that acupuncture at Neiguan (PC 6) can activate the frontal lobe, the temporal lobe, and other brain areas, increase cerebral blood flow perfusion, improve brain blood supply, and promote the recovery of neurological function (38). From the perspective of local anatomy, beneath Jiquan (HT 1) lies the median nerve, the ulnar nerve, and the radial nerve, with both the ulnar and median nerves innervating flexor muscles. For spastic patients, acupuncture should primarily target the extensor muscles innervated by the radial nerve. When the upper limb extensor muscles are stimulated and become excited, leading to contraction, the flexor muscles are inhibited, thereby reducing muscle tone and alleviating upper limb flexor spasticity. Chize (LU 5) is located at the elbow, which is often the most severely affected site for upper limb muscle spasticity. One systematic review summarized the most commonly used acupoints for treating post-stroke spasticity, which included Neiguan (PC 6) and Chize (LU 5), among others (39). Furthermore, the meridian sinew cluster needling involves applying multiple needles along the Yangming Meridian. Multiple needling stimulation along the lateral aspect of the upper limb can extensively activate the nerves of the upper limb, improve neural nutrition, promote metabolic processes in nervous tissue, and enhance its excitability. Simultaneously, it can facilitate local blood circulation, induce contractions in the muscles on the low-tension side of the limb, and increase the strength of the upper limb extensors. Therefore, based on the commonalities of the above conclusions, for patients with post-stroke upper limb spasticity, acupuncture point selection should consider both the medial and lateral aspects of the limb, so as to better coordinate the balance between agonists and antagonists and restore optimal limb movement patterns.

This study has several limitations: (a) Among the 28 included trials, several exhibited suboptimal quality, lacking clear descriptions of key information such as randomization methods, allocation concealment, and blinding. (b) Differences in the selection of acupoints and treatment courses among the included trials may affect the precision of the results. (c) The network graphs did not form closed loops, meaning that efficacy comparisons between many interventions rely solely on indirect evidence. This implies that, although the SUCRA values provide an intuitive reference for ranking the efficacy of different acupuncture methods, the current rankings—particularly the minor differences between adjacently ranked interventions—have low statistical power and limited clinical confirmation. (d) All included studies were conducted in China. Therefore, the applicability of the conclusions in a global context still requires validation through the inclusion of more high-quality studies from diverse regions and healthcare systems.

In conclusion, this study provides a comprehensive assessment of the effects of various acupuncture methods on post-stroke upper upper-limb spasticity through network meta-analysis, clarifying the relative advantages of different acupuncture methods for two outcome measures (FMA and MAS). It offers valuable candidate protocols and priority research directions for clinical practice. However, given the limitations of this study, these findings are not yet sufficient to serve as strong evidence for altering clinical guidelines or practice standards. Future research urgently requires well-designed, rigorously reported large-sample randomized controlled trials, with particular emphasis on the strict implementation and clear reporting of core elements such as randomization, allocation concealment, and blinding. Such high-quality studies are needed to provide direct comparison evidence for verifying or refining the preliminary rankings derived from this network meta-analysis. Until such high-quality evidence is obtained, clinicians should integrate the findings of this study with patients’ specific conditions, clinical experience, and available resources when making treatment decisions.

## Data Availability

The original contributions presented in the study are included in the article/Supplementary material, further inquiries can be directed to the corresponding author/s.

## Supplementary material

SUPPLEMENTARY FILE 1 Detailed search terms.

SUPPLEMENTARY FILE 2 Detailed information on individual studies.
