# Supplementary material for: Network meta-analysis of different acupuncture methods for post-stroke upper-limb spasticity
Source: Front Neurol. 2026 Feb 10;17:1725618. doi: 10.3389/fneur.2026.1725618 (PMC12931285; doi:10.3389/fneur.2026.1725618)
Supplement: SUPPLEMENTARY FILE 1 — Detailed search terms. [file Supplementary_file_1.docx]

Detailed search terms

((((((((((((((((((((Acupuncture[Title/Abstract]) OR (Acupuncture Needling[Title/Abstract])) OR (Xingnao Kaiqiao Acupuncture[Title/Abstract])) OR (Sinew Penetration Needling[Title/Abstract])) OR (Sinew Array Needling[Title/Abstract])) OR (Muscular Region's Acupuncture[Title/Abstract])) OR (Penetrating Needling[Title/Abstract])) OR (Auricular Acupuncture[Title/Abstract])) OR (Abdominal Acupuncture[Title/Abstract])) OR (Scalp Acupuncture[Title/Abstract])) OR (Interactive Scalp Acupuncture[Title/Abstract])) OR (Ocular Acupuncture[Title/Abstract])) OR (Jin's Three Needles[Title/Abstract])) OR (Fascia Originating Points[Title/Abstract])) OR (Giant Needling Technique[Title/Abstract])) OR (Recovery Needling Method[Title/Abstract])) OR (Antagonistic Needling[Title/Abstract])) OR ("Reducing Yin and Reinforcing Yang"[Title/Abstract])) OR ("Harmonizing Yin and Yang"[Title/Abstract])) OR (Balance Acupuncture[Title/Abstract])) OR (Jiaji Points[Title/Abstract])

((((spasm after stroke[Title/Abstract]) OR (post-stroke spasticity[Title/Abstract])) OR (Spasticity after Stroke[Title/Abstract])) OR (spastic dystonia following stroke[Title/Abstract])) OR (((Spastic paralysis[MeSH Terms]) OR ((Spasmodic syndrome[Title/Abstract]) OR (Increased muscular tension[Title/Abstract]))) AND ((Stroke[Mesh]) OR ((((((((Cerebral infarction[Title/Abstract]) OR (Erebral embolism[Title/Abstract])) OR (Cerebral thrombosis[Title/Abstract])) OR (Cerebral hemorrhage[Title/Abstract])) OR (Hemorrhagic stroke[Title/Abstract])) OR (Ischemic stroke[Title/Abstract])) OR (Subarachnoid hemorrhage[Title/Abstract])) OR (Cerebrovascular disease[Title/Abstract]))))

(中风 OR 卒中 OR 脑卒中 OR 脑梗死 OR 脑出血 OR 脑栓塞 OR 脑血栓形成 OR 蛛网膜下腔出血 OR 脑血管疾病 OR 脑梗塞 OR 脑缺血 OR 缺血性脑卒中) AND (痉挛 OR 肌张力增高 OR 痉证 OR 硬瘫 OR 痉挛性瘫痪 OR 痉挛性偏瘫 OR 上运动神经元瘫痪 OR 腱反射亢进 OR 拘挛) AND (针灸 OR 针刺 OR 醒脑开窍 OR 经筋透刺 OR 经筋排刺 OR 经筋刺法 OR 透刺 OR 耳针 OR 腹针 OR 头针 OR 互动式头针 OR 眼针 OR 靳三针 OR 筋膜出发点 OR 巨刺法 OR 恢刺针法 OR 拮抗针法 OR 泻阴补阳 OR 调和阴阳 OR 平衡针 OR 夹脊穴 OR 针) AND (随机对照试验 OR 随机对照实验 OR RCT OR 随机对照研究 OR 随机对照 OR 随机)
